# Towards a coherent global health architecture: perspectives on integrating global health security and universal health coverage through diplomacy and governance reforms

**DOI:** 10.1093/heapol/czaf086

**Published:** 2025-10-31

**Authors:** Arush Lal

**Affiliations:** Department of Health Policy, London School of Economics and Political Science, London, WC2A 2AE, United Kingdom

**Keywords:** global health policy, health systems strengthening, global health security, universal health coverage, coherence, integration, governance, diplomacy, norms

## Abstract

Within the global health landscape exists a complex interplay between global health security (GHS) and universal health coverage (UHC)—two influential agendas with profound influence on health system strengthening initiatives. There is a need to understand why and how coherence between GHS and UHC is being pursued in health policy and planning, particularly in the wake of the COVID-19 pandemic which profoundly reshaped the field of global health and significant cuts to global health assistance. This paper presents one of the first detailed analyses of contemporary efforts to conceptualize and operationalize GHS–UHC coherence—through the perspectives of key actors responsible for its implementation. The study employed thirty-one interviews with senior officials across four major types of global health actor: multilateral and global health organizations, country governments, donors and international finance institutions, and civil society organizations. It reveals important insights into the way specific actor and geopolitical groups varied in terms of shifting perceptions of GHS and UHC, as well as major factors influencing GHS–UHC coherence (e.g. strategic considerations including motivations and concerns, and structural considerations including enablers and barriers). The analysis suggests that an emerging ‘hybrid norm’ linking GHS and UHC appears to be well underway. It further contends that strengthening coherence between GHS and UHC not only depends on, but also enhances, three key imperatives: (i) overcoming geopolitical power asymmetries, (ii) leveraging strategic collaboration across actor types, and (iii) pursuing integrative health diplomacy amid overlapping crises. While this study centres on GHS–UHC alignment, its broader objective is to foster a more equitable and resilient global health architecture by tackling the interconnected causes of fragmentation through hybrid normative frameworks. By focusing on the politics of norms underpinning GHS and UHC integration, this work contributes to rethinking how global health institutions collaborate, ultimately helping to build more sustainable global health governance fit to withstand future political, economic, and social challenges.

Key messages

- Despite recent calls to understand how global health security (GHS) and universal health coverage (UHC) contribute to health system fragmentation, the processes that foster their coherence remain underexplored, particularly how different actors navigate political dynamics and normative alignment to integrate these frameworks in health policy and planning.
- Stakeholders highlighted important differences in how they define GHS and UHC, how they understand the relationship between the two, and the strategic and structural factors they see as most critical for advancing greater coherence between both agendas.
- Strengthening GHS–UHC coherence both requires and reinforces efforts to overcome geopolitical power asymmetries, harness strategic collaboration across actor types, and navigate overlapping crises through integrative health diplomacy.
- The emerging GHS–UHC hybrid norm provides a strategic framework to enhance coordination across global health diplomacy and governance reforms, offering a pathway toward a more equitable, resilient, and coherent global health architecture.

## Introduction

The global health landscape today is characterized by increasing and persistent tension between competing priorities, mechanisms, and institutions. In few areas is this tension more evident than in the complex interplay between global health security (GHS) and universal health coverage (UHC). Both frameworks aim to strengthen health systems and improve public health outcomes, yet their divergent approaches—the former rooted in securitization, the latter in human rights—often result in fragmented governance, disjointed financing, and inconsistent implementation. This incoherence between GHS and UHC undermines equity in health systems, limiting progress toward their shared goal of more resilient populations (Lal et al. 2021).

Significant attempts have already been made to enhance coherence across major global health agendas and initiatives, including by fostering synergies between GHS and UHC (Oliveira-Cruz et al. 2003, Balikuddembe 2020, Tadesse et al. 2021, Agyepong et al. 2023). However, most have fallen short of sustained impact. While related empirical studies employ widely-acknowledged concepts like health system integration (Atun et al. 2010, Cooper et al. 2015) to better coordinate limited resources and harmonize competing health priorities, these often struggle to account for the politics underpinning persistent fragmentation. Drawing on recent calls (Gómez et al. 2022) to recognize the role of ‘political power dynamics’ in explaining ‘why certain public health policies might be more likely to succeed in adoption and implementation’, a focus on how different actors pursue normative coherence between GHS and UHC may provide fresh insights into address this research gap.

This paper presents one of the first detailed analyses of contemporary efforts to conceptualize and operationalize GHS–UHC coherence through the perspectives of key actors shaping their implementation at various levels of health policy and planning. Building on prior work on the historical construction (Lal et al. 2024a) and convergence (Lal et al. 2024b) of GHS and UHC norms, this study examines recent developments towards their integration by engaging with senior officials through in-depth interviews, capturing their insights on the evolving relationship between both frameworks, key factors enabling or obstructing their coherence, and the practical implications for the broader global health architecture. The central research question driving this study asks: how do actors across governments, multilateral institutions, donors, and civil society understand and enact GHS–UHC integration in practice, and what power asymmetries, tensions, and opportunities shape the institutionalization of GHS–UHC coherence at various levels of global health diplomacy and governance?

The conclusions emphasize the emergence of a GHS–UHC ‘hybrid norm’, which both depends on and further enhances: (i) addressing geopolitical power imbalances, (ii) fostering strategic collaboration across diverse actor types, and (iii) pursuing integrative health diplomacy amid today’s era of polycrisis. In doing so, this research seeks to advance broader scholarship on the politics of integrating contested global health agendas in other areas of governance and the role of ‘hybrid norms’ as powerful tools for pursuing collective action and cross-sectoral collaboration in future international initiatives.

### Background

GHS and UHC can be understood not just as important agendas (Smith and Shiffman 2016), but also as influential ‘norms’, each comprised of core ideas, decision-making processes, and organizing principles that shape the behaviour of domestic and international actors (Florini 1996, Finnemore and Sikkink 1998, Lal et al. 2024a). GHS—defined as the activities necessary to mitigate acute public health events that transcend national borders—emphasizes national security and core capacities related to health emergencies (WHO 2007, Stoeva 2020). In contrast, UHC—defined as ensuring all individuals can access a comprehensive range of quality health services without financial hardship—emphasizes the right to health and core capacities related to health equity and primary health care (PHC) (Abiiro and De Allegri 2015, WHO 2025). Examining GHS and UHC as norms, rather than as a set of technical interventions, unlocks new ways of exploring their advancement (and potential for coherence) through greater consideration of underlying principles, values, normative approaches, and obligations on state behaviour (Smith and Rodriguez 2016).

Importantly, this paper does not treat GHS and UHC as coherent or settled concepts. Both are viewed as contested and evolving norms, shaped by competing institutional mandates, political priorities, and actor interpretations, making their integration a deeply political and dynamic process. For example, while this paper foregrounds the rights-based dimensions of UHC—reflecting its discursive positioning in global health diplomacy and high-level declarations—it is subject to dynamic interpretation, often embodying tensions between equity and efficiency or public provision and market-based logics (Giovanella et al. 2018, Rizvi et al. 2020). These contested meanings shape how actors invoke and institutionalize UHC in relation to GHS (and *vice versa*) and underscore the need to analyze normative integration as an ongoing political process.

Scholars argue that over time, GHS and UHC have been (re)constructed as their inherent linkages have become more apparent (Lal et al. 2024a). This suggests that both norms (and their associated ‘regimes’ (Hoffman 2010) or ‘networks of governance’ (Shiffman et al. 2016b), comprised of overlapping institutions and actors, should not be considered as isolated, but rather as dynamic ‘processes’ that continuously evolve as well as influence and reinforce one another. Therefore, while GHS and UHC are each contested and evolving norms in their own right, the interactions and convergence between them—particularly in the context of crisis response (e.g. COVID-19 pandemic), high-level diplomacy, and institutional mandate expansion—suggest the early emergence of what might be termed a ‘hybrid norm’.

Representing the partial and interlinked pursuit of two previously-distinct norms, hybrid norms can be seen to capture growing complementarity and interdependency between the underlying discourse and core functions of two or more parent normative regimes (Lal et al. 2024b). This does not imply a fully crystallized or standalone ‘hybrid norm’ with universally-agreed components. Rather, hybrid norms refer to dynamic normative formations that arise through the ongoing negotiation, adaptation, and partial integration of existing normative regimes.

This framing builds on constructivist scholarship on norm interaction (Krook and True 2012, Wiener 2018) and norm complexity (Fehl 2019, 2020), which examine how norms coexist, conflict with, or reinforce one another across institutional settings. Hybrid norms extend this literature by emphasizing the emergent blending of normative elements (e.g. problem framings, behavioural expectations, and governance mechanisms) across distinct regimes. As Winston highlights, norms are structurally flexible and can evolve through recursive interaction (Winston 2018). This article therefore treats the GHS–UHC hybrid norm not as fully formed, but as an evolving configuration whose boundaries are still being shaped through practice and diplomacy.

The uptake of a hybrid norm promoting both GHS and UHC is increasingly manifest across recent developments in global health governance, financing, and programming—from multilateral health resolutions (WHO 2025) to government strategies (FCDO 2021), to new initiatives like the Lusaka Agenda (FHGI 2023) and the Accra Reset. Given the profound influence of both frameworks on shaping health systems strengthening and broader global health efforts, previous research has attempted to advance GHS and UHC synergies, including through case studies unpacking ‘multiple interconnected factors causing fragmentation at the global level’ (Agyepong et al. 2023). However, without analyzing the politics of norms in shaping why and how actors choose to collaborate on different global health agendas, these studies have overlooked key insights into power dynamics and institutional behaviours vital to sustaining normative coherence, particularly in a post-pandemic landscape where both GHS and UHC have undergone significant conceptual shifts.

To reiterate, this paper treats the hybrid norm linking GHS and UHC as emergent—not yet fully codified or named, but increasingly visible in the convergence of problem framings (e.g. resilient primary health care), institutional mandates (e.g. WHO’s GPW13), and actor discourses around integration. The aim, therefore, is not to assert the existence of an altogether new, discrete norm, but to trace how actors involved in global health diplomacy perceive and shape coherence across the GHS–UHC divide, and what normative logic may be consolidating through these interactions.

Recognizing that opportunities are ‘created when demonstrable synergies and benefits can be achieved by integration’ (Atun et al. 2010), this analysis draws on collective action frameworks theoretically-grounded in political science and international affairs (Shiffman et al. 2016a, Smith and Rodriguez 2016)—particularly the concept of ‘policy coherence’, which is characterized by ‘mutually reinforcing policy actions’ across ‘different types of public policies, between different levels of government, between different stakeholders and at an international level’ (OECD 2023). While not used to explicitly structure data collection or coding, these theoretical concepts provided a useful lens to interpret how actors conceptualize coherence and navigate institutional integration across both agendas.

By advancing scholarship on hybrid norms in global health, this paper importantly places the analytical emphasis on what Barnett and Duvall termed ‘constitutive relations’—‘the social processes that define the identity of actors and their relationships, with consequent effects on what these actors can do’ (Gómez et al. 2022). This study therefore further contributes to systems thinking in health policy (Kwamie et al. 2024), positing that GHS–UHC coherence can foster necessarily integrated global health solutions amid interrelated external threats and geopolitical headwinds.

## Methods

### Data collection

Between May and July 2024, a total of 31 in-depth, semi-structured interviews were conducted with key informants across four institutional types: multilateral and global health organizations, country governments, donors and international finance institutions, and civil society organizations. All actors were selected for their active role in the governance of global health initiatives and diplomacy, particularly in the response to COVID-19 and ongoing health system strengthening (HSS) efforts. Together, these groups represent the diverse and often divergent views influencing GHS and UHC—actors whose decisions are shaped not only by technical considerations, but also by power, influence, and politics—offering vital lessons for fostering policy coherence across various global health institutions, donors, and practitioners. The timing of this study is significant, given major reforms to global health architecture launched after the COVID-19 pandemic and sparked by stocktaking at the midpoint of the 2030 SDGs deadline, when discussions about coherence between GHS and UHC have intensified.

The sampling strategy was designed to reach senior officials directing efforts in GHS, UHC, and/or HSS at major global health institutions. This was done using a combination of purposive sampling and snowball sampling to reach relevant participants (see Tables 1 and 2). Careful consideration was taken to ensure a relative balance across actor type as well as Global South (GS) versus Global North (GN) representation to provide unique insights into GHS–UHC coherence based on varying institutional and geopolitical dynamics (Sriram et al. 2018, Topp and Topp 2020).

**Table 1.** Number of key informants interviewed per actor grouping

Actor type	No. of participants
Multilateral and global health organizations	9
Country governments	10
Donors, foundations, and international finance institutions	6
Civil society organizations	6
Total	31

**Table 2.** Number of key informants interviewed per geopolitical grouping

Geopolitical group	No. of participants
Global South	13
Global North	18
Total	31

### Data analysis

To analyze the data, Braun and Clarke’s systematic approach to thematic analysis (Braun and Clarke 2006) was employed, beginning with repeated readings of the transcripts to ensure familiarity with the narratives and identify recurring ideas and contradictions. NVivo software was used to develop and refine codes both inductively from the data and deductively based on the research focus and interview questions. These codes were iteratively grouped into subthemes, themes, and broader categories to structure the results section and align with the study’s aim of unpacking the politics of GHS–UHC coherence. To ensure robustness, findings were triangulated by cross-referencing interview data with field notes and global health policy documents, enhancing credibility and minimizing bias. Therefore, while the thematic analysis was primarily inductive, with codes generated iteratively based on patterns observed across interviews and documents, prior literature on global health governance and policy coherence informed the interpretive framing of findings, particularly in identifying how actors understand tensions, trade-offs, and integration strategies. This rigorous, iterative approach effectively synthesized diverse stakeholder perspectives, revealing previously-overlooked power dynamics that shape GHS–UHC coherence and political economy implications of hybrid norms within the post-COVID-19 global health landscape.

## Results

The findings from the elite interviews are structured into two overarching categories: evolving perceptions of GHS and UHC (definitional shifts and conceptual relationships), and key factors influencing coherence (strategic and structural considerations). Each category is broken down into themes and subthemes, which highlight emerging insights and direct quotes from participants (see Table 3).

**Table 3.** Findings from thematic analysis of interview transcripts, organized by category, theme, and subtheme

Category	Theme	Subtheme
Evolving perceptions of GHS, UHC, and their relationship	Definitional shifts	GHS emphasis on equity and community-level health services UHC as key to resilience, despite recent deprioritization
	Conceptual shifts	UHC as foundation for GHS Interlinked through health system
Factors influencing coherence	Strategic considerations	Motivations Improving health outcomes Maximizing efficiencies Sustaining progress for both Concerns Harder to demonstrate progress Internal resistance and competition Weakened messaging
	Structural considerations	Barriers Conceptual and structural misalignment Power dynamics and external influences Resource allocation and accountability Enablers Clear messaging External support Internal implementation

### Definitional shifts

#### GHS emphasis on equity and community-level health services

The COVID-19 pandemic prompted a paradigm shift in the perception of GHS, with increasing emphasis on equity and the continuity of community health services.

One GN civil society advocate articulated this evolution: ‘*Health equity is a big component of global health security, which I don’t think necessarily falls in the standard definition. But security is about protection, right? And so that’s protection for all people*’.

While GN actors often focused on aligning GHS with global strategies, GS voices emphasized new imperatives for grounded, bottom-up approaches that resonate with communities. For example, a GS civil society representative argued, ‘*Global health security must be solved in communities […] there is no security in global health if there is no security in communities*’.

#### UHC as key to resilience, despite recent deprioritization

Although the pandemic underscored UHC as a foundation for GHS, it also diverted political attention and financial resources away from UHC efforts, with a former GN government official lamenting that UHC was ‘*forgotten [and] deprioritized*’. A GN civil society advocate further highlighted:

‘*Unfortunately for the UHC movement, it was just getting its legs when COVID-19 hit […but…] if you don't have a strong health system that you built through UHC or have people being able to access the system, pandemic response doesn't really work*’.

Meanwhile, GS actors emphasized UHC’s role in supporting resilience, with a multilateral official arguing that, in countries facing frequent shock events, ‘*you cannot achieve UHC, because you are draining your resources on fighting the fire in lieu of making your house fire-protected*’.

These definitional shifts reflect the dynamic, contested nature of both GHS and UHC as evolving norms, supporting constructivist claims that norms are socially constructed and reinterpreted in response to shifting crises, institutions, and actor priorities.

### Conceptual relationships

#### UHC as a foundation for GHS

One way that key informants frequently conceptualized the relationship between both goals was by arguing that UHC was a broader framework, under which GHS was one element.

As one GN government official explained, ‘*global health security is a part of the overarching universal health coverage framework […] if done correctly, [UHC] can mitigate the need for a whole bunch of security efforts*’.

Several GS actors agreed, with one donor stating, ‘*there is no way you can get global health security without universal health coverage*’, and a former GS government official suggesting, ‘*what comes first is universal health coverage. That's the big umbrella for anything else. I see global health security within that*’.

#### Clear interrelatedness between GHS and UHC

The post-pandemic era also catalyzed greater recognition of the interdependence between GHS and UHC, with stakeholders increasingly viewing them as complementary norms.

Two multilateral officials encapsulated this best, with a GN-based representative remarking that their ‘*relationship is unavoidable and is fully interdependent*’, and a GS-based counterpart reflecting: ‘*the two constructs are intertwined, interlinked via the health system […] they are two goals*’.

This theme speaks directly to norm complexity literature, which emphasizes that apparent normative incoherence often stems from overlapping but unresolved normative logics rather than technical disagreement alone.

### Strategic considerations: concerns for increasing GHS–UHC coherence

#### Harder to demonstrate progress

Integrating GHS and UHC presents challenges in relation to GN actors’ reliance on tangible outcomes for funding justification. One GN donor representative noted:

‘*You can rattle off a list of community health workers and surveillance […] where it feels there is overlap between UHC and pandemic preparedness […] but whether that is genuinely tracking through to the way in which work is being […] planned and done is a very different question*’.

GS actors, in contrast, emphasized the need for equity-focused metrics to ensure that marginalized populations are not left behind during integration efforts, with a civil society representative pointing out: ‘*you could overlook certain elements […] when you try to talk about UHC […and…] global health security […] You don’t have indicators for who is covered*’.

#### Internal resistance and competition

Resistance to integrating GHS and UHC appeared to stem from entrenched institutional structures and competition for resources, with one GN government official arguing that ‘*people do not want to understand or collaborate because [they] are very keen to deliver on their own agenda*’. A GN donor further reflected:

‘*People’s jobs exist to perpetuate the status quo. The global health institutions (GHIs) have been designed this way. Ministries of health have been redesigned to interface with that […] whilst there may be theoretical high-level support for integration, people’s individual jobs and incentives often don’t align with that*’.

A GS health multilateral echoed: ‘*You have a small pot of money, […] as we talk about harmonizing, we also need to talk about bringing […] the stakeholder groups that are purposely fighting for specific disease areas together*’. A GS government official further explained how ‘*too many cooks in the kitchen*’ fuels unnecessary competition, already exacerbated by the exclusionary dynamics of global health governance.

#### Weakened messaging

Overall, GN actors of all types emphasized the dilution of clear, actionable narratives necessary to sustain political and financial support as a primary concern for GHS–UHC coherence.

One GN government official observed:

‘*I think your message gets diluted. […] single disease conversations have been so successful, like HIV, Malaria, TB. Because you’re really just focusing on one thing. And that seems very solvable. Once you […] keep expanding out, it becomes overwhelming […] that’s also why GHS as a standalone thing has been very captivating […] because it seems very tangible*’.

Meanwhile, GS actors were concerned about possible erosion of the specific priorities and principles of each framework, with one civil society representative warning, ‘*how do we ensure that even as we integrate, we don’t lose the unique vulnerabilities […] around GHS? And UHC?*’

This divergence highlights a tension between GN actors’ focus on the clarity of individual messages versus GS actors’ insistence on maintaining a comprehensive view of both norms. These concerns reflect the constitutive effects of global health governance arrangements, in which institutional identities and role expectations often shape what actors perceive as legitimate or feasible forms of integration.

### Strategic considerations: motivations for increasing GHS–UHC coherence

#### Improving health outcomes

Stakeholders consistently identified the integration of GHS and UHC as essential for improving health outcomes, particularly due to greater coordination and resilient health systems capable of maintaining essential services during crises.

One GN civil society representative asserted, ‘*if we invest in UHC, if we invest in health security, […] that will lead to fewer deaths and better economic outcomes, better health outcomes, […] better societal outcomes.’* A GN government official similarly emphasized better outcomes ‘*if we’re more coordinated across the various [GHS and UHC] programmes*’.

However, GS actors sometimes went further than their GN counterparts to highlight the compounding effects of health crises which may ‘*driv[e] people into poverty*’, with one civil society representative stressing that, ‘*equity […] is a fundamental reason why [GHS and UHC]*’ must be integrated.

#### Maximizing efficiencies and resources

The integration of GHS and UHC was frequently highlighted as an opportunity to optimize resources and reduce inefficiencies, particularly by harmonizing health systems components.

One representative of a GN-based donor observed:

‘*We are duplicating funding infrastructure […] we don’t […] understand where the overlaps and opportunities are to streamline, to mainstream, to de-fragment […] it’s increasingly […] about how we can do better with the resources out there.*’

Meanwhile, a GS donor noted:

‘*If you address health security as something completely distinct from universal health coverage, then your actions [are] not properly synchronized on the ground, you are just wasting resources […] synergies help in efficiency, help in bringing the results faster.*’

GS actors’ emphasis on the risk of wasted resources and fragmented attention on the ground indicates a pragmatic focus on avoiding operational inefficiencies that directly affect service delivery—a slight nuance compared to GN counterparts.

#### Sustaining progress for both agendas

Various stakeholders were motivated by the potential to sustain advancements for both GHS and UHC, largely because they believe initiatives that build coherence can mitigate donor fatigue, prevent the creation of new silos, and amplify the visibility of both agendas through a shared platform for advocacy and funding.

One GN donor cautioned against conventional vertical programmes that ‘*cause fragmentation*’, with a GN government official emphasizing joint/pooled initiatives by noting that ‘*strength in numbers is the important thing*’.

With actors across the board agreeing that ‘*global financing for health is going down*’ a GS donor asserted that without synergizing GHS and UHC, ‘*you are dispersing attention and you will never reach your goals*’—underscoring the importance of aligning funding streams. This demonstrates a shared interest among GN and GS actors toward sustainability, but a divergence in emphasis on operational versus financial priorities to sustain progress, with a need to balance top-down strategies with bottom-up approaches to ensure effectiveness.

These accounts help illustrate how integration may employ a form of normative entrepreneurship where actors selectively mobilize coherence to advance organizational mandates and global positioning.

### Structural considerations: barriers to GHS–UHC coherence

#### Conceptual and structural misalignment

Divergent conceptualizations between GHS and UHC frameworks emerge as significant barriers to coherence, reflecting unique priorities and approaches among different actors.

A central reason for the disconnect may be attributed to the conceptual divide in how GHS and UHC are framed, as expressed by a GN government official: ‘*UHC is about local, country-specific goals, while GHS requires cross-country collaboration’.* This dynamic may be compounded by ideological divides, with another GN government official suggesting: ‘*The GHS agenda is more right-wing, while UHC is more left-wing’,* reflecting competing politics that hinder collaboration.

This conceptual misalignment perpetuates silos between GHS and UHC, with each stream accountable to different mechanisms, as noted by a GS-based civil society advocate: ‘*Each global health institution and pot of money has its own specific mandates’.* One GN-based public–private partnership similarly noted: ‘*Structures in place for financing global health are disease-specific, technology-specific, and population-specific […these] define how the money is raised, who pays and where the money goes’.*

GS actors have described how this fragmentation also hinders country-level implementation, with one civil society representative pointing out: ‘*the pandemic preparedness and response (PPR) team and UHC team are often siloed within countries’.* Donor-driven systems, they argue, thus create fragmented pathways that are poorly aligned with the integrated vision required for coherence. This bifurcation reveals a deeper issue of divergent operationalization of GHS and UHC initiatives within different actors.

This leads to variation in how both concepts are communicated, with one GN donor highlighting that GHS and UHC ‘*discourses […] happen in separate parallel tracks where, in fact, they ought to be combined*’. Civil society actors emphasized that GHS’s simplicity—‘*detect an outbreak, contain it, and prevent global spread’*—makes it more palatable to policymakers than UHC’s multidimensional complexity, a sentiment echoed by a GS multilateral official who observed that UHC is often dismissed as ‘*aspirational’* by practitioners focused on more ‘immediate’ public health challenges. GN actors therefore focused on aligning overarching narratives and funding streams, while GS voices stressed the importance of tailoring integration efforts to local realities.

#### Power dynamics and external influences

GS donors described a clear imbalance in power and influence, with one observing, ‘*The game always is that those who have [more money] take advantage and impose their ideas*’. Many informants argue that this leads to an absence of GS voices in shaping the global health agenda, with low- and middle-income countries (LMICs) often lacking leverage and coming to the table in what one GS multilateral official characterized as a ‘*pity party dynamic’.*

Power asymmetries between GN and GS actors (and across institutional types) therefore hinder GHS–UHC coherence, with GS stakeholders frequently perceiving GN-driven frameworks as misaligned with local needs, such as another GS multilateral representative noting that: ‘*global health security is often construed with a very global north lens*’.

Of note, some GN actors did acknowledge these tensions, with one government official contending:

‘*The ways of doing development really need to change […] if there's a plan towards UHC or there's a plan towards improving health security in a country, we need to be thinking about how our work can fit into what they have proposed*’.

However, a GS regional health agency representative argued that some of the solutions lie closer to home:

‘*If regional organizations need funding, it’s easy for them to be swayed by what a global player says. […] we need strong institutions with strong leaders who […] know what their priorities are, to align global level financing to what they believe is the priority of the country*’.

### Resource allocation and accountability

Fragmented funding mechanisms and insufficient accountability structures exacerbate barriers to GHS and UHC integration, revealing significant disparities between GN and GS actor groups.

Donors often focus on narrow, vertical funding mechanisms, as one GN civil society representative observed a ‘*lack of long-term vision on the part of funders […] people fund a report, a project, an event […] but not building a movement […] for both agendas that are quite broad and long term’.*

GS actors face additional challenges in securing sustainable financing. One GS representative observed ‘*in many countries […] sufficient resources is not there. And then they resort to institutions like the Global Fund’,* which they argue inherently prioritize donor-driven priorities over local needs. This imbalance undermines national health systems, with one GS donor describing ‘*the draining of staff from the public sector to go to the programmes which were funded by HIV and AIDS programmes because they had money*’ and better working conditions.

This piecemeal approach undermines clear accountability mechanisms, with one GN government official cautioning that ‘*there’s no legislation […] there’s got to be coordination across these various departments and agencies’.* A GN-based multilateral representative further urged: ‘*we have to address [GHS and UHC] as an integrated way and in the health system […] what is lacking here is who is the policeman […] who secures the accountability*’.

These barriers underscore the political dimensions of fragmentation and institutional path dependency, highlighting how rigid architectures and siloed financing structures constrain efforts toward coherence.

### Structural considerations: enablers for GHS–UHC coherence

#### Effective communication

Effective communication emerged as a critical enabler for integrating GHS and UHC, though GN actors seemed to emphasize this more than their GS counterparts. One GN government official highlighted the importance of crisp, tailored messaging:

‘*A policymaker […] wants something explained to them in a page or less […] when you're trying to change hearts and minds […] how do you focus the conversation in a way that resonates with them?*’

This stands in contrast to the perennial challenge of succinctly advocating for cross-cutting initiatives required for HSS or GHS–UHC coherence, with a GN civil society representative explaining: ‘*we know that complexity leads to confusion, which leads to less buy-in and investment*’.

Several informants suggested that more senior, political officials may be better accustomed to viewing issues through integrated ‘big-picture’ perspectives, rather than distinguishing between GHS and UHC; coherent narratives may therefore resonate better with them than with lower-level technocrats. For example, one GN-based donor observed: ‘*the Prime Minister is not going to say,* ‘*Oh, you're coming to me on a health security issue today. you're coming to me on a UHC issue […they] see health as health*’.

A senior GS multilateral official echoed this sentiment, and its implications for GHS–UHC operationalization:

‘*Who is running a health service in some of the most challenging environments? It’s the same person. The Director-General of Health Services may be responsible for setting out a PHC strategy, an overall national health policy […] overseeing the National Action Plan for Health Security. […] one plan, one budget, one monitoring—that has to be the binding factor*’.

Part of the solution may therefore be in communicating GHS and UHC in more personal ways, with one GN multilateral representative stating, ‘*we need to still bring these people, these [GHS and UHC] groups together […] it’s a cultural integration*’.

### External support

External support through partnerships, collaboration, and funding mechanisms was seen as a critical enabler for GHS-UHC coherence—with relatively converging views across all actor types on streamlining funding channels and fostering synergy among institutions.

One GN multilateral health official remarked, ‘*the funding flows need to work towards that objective. We now have vertical funds that try to do more horizontal stuff […] but these funds are certainly in competition with one another. But it’s the same health system you're trying to strengthen*.’ A GS-based civil society advocate further argued:

‘*Look at the global health institutions that are already supporting countries to strengthen health systems […] when the pandemic hit, the Global Fund was one of the first partners to put money on the table to respond so rapidly […] How can all these different organizations synergize? It’s about collaboration, coordination*’.

In response, one GN-based donor emphasized: ‘*The spirit of the Lusaka Agenda and the Future of Global Health Initiatives (FGHI) process that led it was very focused on what are the priorities at country level and how can we get the global financing system to better align and interface with those*’. Many actors believed this might catalyze coherence, given that domestically, ‘*these things are all incredibly enmeshed. You cannot separate out surveillance for global health security from routine health monitoring and system strengthening*’.

### Internal implementation

Domestic prioritization and commitment to health—reflected in national budgets and through leadership—were consistently highlighted as critical to GHS–UHC coherence, though the framing differed across GN and GS actors.

One GN government official noted clear value-for-money if efforts for GHS–UHC coherence can secure necessary political leadership:

‘*If Ministries of Health are […planning…how…] money for GHS can be used in conjunction with UHC principles, then […] it’s such a self-evidently smart argument […] there has to be a desire by the national government to actually do this, to appoint [and empower] leadership […] and then to convene stakeholders, multilateral organizations, funding institutions, bilateral partners*’.

Meanwhile, GS actors focused more on national capacity-strengthening and resilience that could catalyze GHS–UHC coherence. For example, one GS official of a global health agency observed: ‘*If you look at national-level planning […] all of these are particular silos. One easy fix is to bring all these plans into one integrated plan. It's doable, but it's not happening*’. A former GS government official further concluded, ‘*we need to advocate for a holistic, multisectoral, coherent approach to governance*’.

This theme indeed aligns with existing scholarship on policy coherence, which stresses the importance of institutional coordination, high-level political will, and mutually-reinforcing mandates as key enablers of normative alignment.

## Discussion

While the shift towards integration of GHS and UHC is not entirely new (Lal et al. 2024a, 2024b), the COVID-19 pandemic provided a clear policy window (Weber and Driessen 2010) that increased its visibility and urgency, reinforcing the need for a coherent approach combining emergency response with equitable access. Crucially, all respondents (*n=* 31) believed enhancing GHS–UHC coherence was an important endeavour, with many emphasizing the need to address fragmentation and identify opportunities for advancing integration, including in their own organizations. This represents an important normative advancement, indicating that socialization (Finnemore and Sikkink 1998) of an emerging ‘hybrid norm’ linking GHS and UHC is clearly underway.

Analysis of the overarching themes suggests that strengthening coherence between GHS and UHC not only depends on, but can further enhance, three key imperatives in health policy and planning: (i) overcoming geopolitical power asymmetries, (ii) leveraging strategic collaboration across actor types, and (iii) pursuing integrative health diplomacy amid a polycrisis. Moving forward, the emerging hybrid norm of GHS–UHC integration can be used to identify best practices and priority actions for how global health governance structures, financing mechanisms, and health systems strengthening initiatives are designed, particularly in a broader diplomatic context characterized by calls for coherence in the face of geopolitical headwinds and resource constraints.

### Overcoming geopolitical power asymmetries

The findings reveal entrenched power asymmetries in global health governance, where GN actors dominate agenda-setting, financing decisions, and programmatic priorities—often sidelining GS perspectives in shaping long-term health policies. Nguenha and colleagues note that ‘part of the challenge of coherence across sectors’ is that stakeholders perceive the same issues through vastly different lenses (Nguenha et al. 2024). This divergence is evident in how GN donors prioritize vertical, outcome-driven investments favouring GHS-aligned initiatives such as HIV/AIDS, tuberculosis, and malaria, while GS governments—contending with the dual burden of infectious and chronic diseases and inadequate health services—emphasize UHC-driven capacity-building and primary health care. Such asymmetries, reinforced by unequal decision-making power (Kickbusch and Liu 2022) and misaligned resource allocation, perpetuate conceptual and operational divergences that hinder efforts to sustainably address complex health challenges. However, a hybrid norm linking GHS–UHC provides a strategic pathway to reshape the global health agenda in ways that are mutually-beneficial for GN and GS actors. For example, integrating UHC’s emphasis on equitable access within GHS’s emphasis on emergency response, could redefine financing objectives, incentivize joint investments, and create mechanisms for more inclusive governance (i.e. UHC principles like equity and universality may help reframe emergency response as an issue requiring holistic coverage and affordable services, not just outbreak control—thereby encouraging pooled investments in PHC as well as platforms like the Pandemic Fund to include LMIC priorities and community delivery models). In this way, advancing GHS–UHC coherence can usher in structural realignment in multilateral engagement, positioning GS countries as active architects—rather than passive recipients—of global health architecture reforms that reflect their priorities.

As Fidler argues, the transition to a ‘multipolar’ world (Fidler 2023) necessitates new frameworks that balance the geopolitical interests of middle-income countries with evolving global health governance. The hybrid norm of GHS–UHC helps achieve this balance, promoting cross-cutting principles and ways of working that help shift influence from GN actors to regional bodies and geopolitical blocs with stronger GS representation. As Riggirozzi’s notes, regional entities play an ‘important role in advancing health agendas in member countries’ by offering more equitable governance structures (Riggirozzi 2017) and may therefore be uniquely positioned to operationalize this shift. The African Union’s vision for a ‘new public health order’ (Africa CDC 2023) underscores the need to embed GHS and UHC within treaty mechanisms and institutional structures to ensure long-term sustainability, while BRICS—leveraging its growing geopolitical influence (Tediosi et al. 2016)—can drive alternative health financing models that challenge GN’s conventionally neoliberal, vertical health reforms. At the same time, shifting global dynamics may open new pathways for GS actors to redefine health priorities through integrative frameworks. For example, with the second Trump presidency significantly gutting global health programmes (Burki 2025) and major donors slashing official development assistance (ODA), GS actors should increase public financing for domestic health programmes, reshaping priorities by positioning UHC as a central pillar and reconceptualizing GHS through more equity-driven and sustainable approaches (Kickbusch and founder 2024). Finally, advancing GHS–UHC coherence can help bridge broader economic and foreign policy agendas—including trade and development finance—enabling collective action on socioeconomic determinants of health through key platforms like the G20 Health-Finance Task Force, and reduced reliance on externally-imposed priorities through improved South–South collaboration. If strategically leveraged, GHS–UHC coherence could mark a turning point in global health governance by striking a fairer balance between solidarity and health sovereignty – ensuring that regional partnerships (Rahman-Shepherd et al. 2025) evolve through integrative frameworks (rather than siloed interventions) to shape the next era of multilateral health cooperation.

The Lusaka Agenda’s focus on country ownership represents a critical shift in global health governance, aiming to correct longstanding imbalances in decision-making and financial flows that have historically prioritized GN donor-driven agendas over GS national strategies. However, past efforts to reform donor coordination—such as the Paris Declaration on Aid Effectiveness (Buse and Walt 1996)—have failed due to persistent structural barriers, including fragmented institutions and a reluctance to relinquish control over resource allocation. Here too, the emerging GHS–UHC hybrid norm provides a conceptual and operational framework that could help the Lusaka Agenda and Accra Reset (Ofosu 2025) as transformative tools for proponents to bridge the divide between externally-driven priorities and locally-led HSS efforts through a mutually-reinforcing policy paradigm. Furthermore, positioning GHS and UHC as coherent, interdependent objectives, rather than competing priorities, could incentivize harmonized investments that respect country ownership while ensuring that global health priorities remain responsive to both domestic and transnational health challenges. Policymakers may consider operationalizing this approach in a forthcoming World Health Assembly Resolution or UN General Assembly Declaration, as part of broader efforts towards global health architecture reform.

Finally, GHS–UHC coherence advances efforts to decolonize global health (Abimbola et al. 2024 , Baum et al. 2024) by reconfiguring GN–GS engagement, shifting from short-term aid dependency (e.g. official development assistance) to long-term structural investments in HSS (Sridhar *et al*. 2008, Sparkes *et al*. 2024). This transition requires more than rhetorical commitments—it necessitates dismantling entrenched financing conditions and decision-making asymmetries that perpetuate GN dominance (Pai et al. 2024). Interviewees highlighted that GHS–UHC coherence offered a pragmatic mechanism in the wake of COVID-19 for reconciling GN priorities in surveillance and intelligence-sharing with GS demands for equitable access to countermeasures and sustainable health financing. Beyond pandemic response, such hybridization ensures that equity is not merely an aspirational principle but an operational imperative for resilient health systems. By embedding coherence within global governance frameworks, this approach fosters mutual accountability, aligns incentives across geopolitical divides, and promotes an inclusive, sustainable model of global health policy that resonates with both GN and GS actors alike.

### Leveraging strategic collaboration among different actor types

Advancing GHS–UHC coherence necessitates leveraging the distinct, yet complementary, roles of governments, multilaterals, donors, and civil society—while dismantling entrenched institutional barriers and path dependencies (March and Olsen 1998, Raymond et al. 2014, Lal et al. 2024b). As Lencucha argues, ‘bureaucratic silos’ (Lencucha et al. 2018) between these actors continue to obstruct coordination, with respondents underscoring inefficiencies, competing priorities, misaligned funding cycles, and institutional resource guarding as primary obstacles. Historically, governance structures have reinforced these divides, with narrowly defined mandates that have made interests resistant to integration. The emerging GHS–UHC hybrid norm offers a mechanism to navigate these challenges—not merely by harmonizing actor roles, but by establishing clear normative pathways for information-sharing, aligning financial incentives, and fostering multi-sectoral accountability. For example, GHS actors often bring surveillance capacity and vertical funding, while UHC actors anchor inclusive governance and equity goals; coherence between both norms can promote shared dashboards (e.g. Joint External Evaluations/Service Availability and Readiness Assessments alignment), joint budgeting, and mutual accountability frameworks across ministries and donors. By addressing the “lack of shared information, delayed and ineffective decision-making as well as the inability to resolve ‘wicked problems’” (Quintana et al. 2024), GHS–UHC coherence may therefore enable a structural shift toward strategic collaboration. However, its success depends on sustained political will and institutional buy-in, requiring deliberate efforts to embed coherence into governance frameworks, funding structures, and long-term planning.

Governments are central to national health resilience, tasked with responding to public health threats while ensuring equitable access to care. The emergence of a GHS–UHC hybrid norm reinforces this dual mandate by fostering a whole-of-government approach that integrates essential public health functions with primary health care (Lal and Schwalbe 2023), strengthening institutional capacity beyond crisis response. Respondents emphasized that advancing GHS–UHC coherence requires prioritizing cross-cutting health systems interventions, including sustained investments in health workforce development, information systems, and public health infrastructure. The ability of GHS and UHC to bridge ‘sectoral differences’ (Quintana et al. 2024) is particularly critical for overcoming fragmented health policies across ministry agencies and mitigating the effects of electoral cycles (Siirilä and Salonen 2024), which often hinder long-term strategic planning. Furthermore, interviewees from across actor groups underscored the importance of governments actively championing GHS–UHC integration both domestically and internationally, leveraging diplomatic channels to align global health agendas with national priorities, and advocating for greater domestic health budgets.

Multilateral institutions and global health organizations are central to fostering coherence between GHS and UHC, providing normative, technical, and coordination functions across the global health architecture. Their effectiveness in harmonizing health guidance, aligning fragmented health financing flows, and promoting synergies across overlapping agendas, however, remains constrained by competing replenishment cycles, earmarked funding streams, and institutional governance structures that reinforce fragmentation. Many stakeholders underscored the need for a normative shift within these institutions, beginning at the highest levels of leadership—particularly among governing boards of public–private partnerships, where decision-makers remain largely unaccountable (de Bengy Puyvallée 2024)—and extending to country offices and community leaders. The hybrid norm of GHS–UHC may, in turn, provide a framework for balancing immediate outbreak responses with long-term HSS (Spasenoska et al. 2025), equipping policymakers and technical specialists with integrated metrics such as WHO’s revised International Health Regulations (IHR) benchmarks and Health Emergency Preparedness, Response and Resilience (HEPR) framework (WHO 2023) to enhance accountability across global health programmes.

Atun *et al*. argue that “fiduciary requirements imposed on donor agencies by their governing structures which require them to ‘ring fence’ funding streams or be able to attribute results to their investments” significantly hinder integration (Atun et al. 2010). Interviewees echoed this concern, emphasizing that GHS–UHC coherence could be advanced through aligned funding mechanisms and pooled, multi-year investments, reinforcing broader literature that underscores the need for more flexible, adaptive financing models (Atun et al. 2010, Yates 2021, Lal et al. 2022a, Holmer et al. 2025). The norm linking GHS and UHC offers a strategic lens through which donors can reduce duplication, maximize synergies across overlapping priorities (Sachs et al. 2022), and support localization efforts (Charani et al. 2022), ultimately enhancing the cost-effectiveness and sustainability of global health investments. However, respondents warned against new vertical funds or flashy initiatives that exacerbate fragmentation, instead advocating for strengthening existing funding channels and prioritizing horizontal financing. For example, the Pandemic Fund could be strengthened by aligning with GHS–UHC coherence, ensuring that all future investments serve a dual-purpose of strengthening pandemic preparedness alongside universal access to care and equitable health system resilience.

The findings highlight the critical role of civil society organizations in advancing GHS–UHC coherence, as they are uniquely positioned to contextualize global health policies within local realities, such as ensuring that frontline health workers with a focus on vulnerable and marginalized groups remain central priorities. Their role in governance accountability is well-documented (Smith 2019 , 2023), with a strong emphasis on inclusivity, community engagement, and coalition-building (Rau 2006, Olu et al. 2019, AlKhaldi et al. 2021, Ngongo et al. 2024), bridging high-level global health strategies with implementation at national, subnational, and community levels. However, respondents underscored persistent challenges, including resource constraints and pervasive power imbalances, which often hinder the ability of civil society organizations to shape policy. Greater investment in civil society-led initiatives could strengthen the operationalization of an emerging GHS–UHC hybrid norm, which in turn would enable sustained advocacy for both GHS and UHC across shifting investment landscapes, while fostering the trust, political will, and multistakeholder collaboration essential for equitable and inclusive health systems.

This study advances scholarship on the politics of integration (Storeng and Béhague 2016) by demonstrating how normative coherence between GHS and UHC can enhance collaboration across diverse actors. Shifting from fragmentation’s empirical drivers to actively fostering hybrid norms offers a pathway to overcoming institutional competition for resources and attention, longstanding barriers to equitable global health partnerships (de Bengy Puyvallée et al. 2025). Strengthening GHS–UHC coherence transforms competing agendas into synergies, reinforcing more resilient and responsive health systems amid urgent calls to reform the global health architecture (Rasanathan et al. 2025, Saleh et al. 2025). While divergent perspectives remain a challenge, aligning these frameworks enables actors to leverage their strengths in governance, financing, health systems, and political mobilization to drive more unified and sustainable global health strategies.

### Navigating a polycrisis through integrative health diplomacy

The findings emphasize the urgency of systemic, integrated responses to complex global health challenges that transcend siloed approaches. The concept of a polycrisis (Wong et al. 2024) characterizes today’s era where pandemics, climate change, and armed conflicts intersect with economic fragility, rising authoritarianism, and the erosion of multilateralism, threatening to stall or reverse public health gains. This fragmented landscape poses formidable challenges for global health cooperation, which ‘must be viewed through the lens of systemic risk’ (Kwamie et al. 2024), requiring multifaceted responses that cross sectors and borders. As the 2030 SDG deadline nears, resilience and the ability to mitigate emerging, overlapping threats must become central to the ‘everyday business’ of health systems (Rasanathan 2024). To navigate this landscape, coherent and adaptive health strategies are essential, not only to withstand crises but to transform global health governance into a system capable of anticipating and responding to compounding risks.

Kickbusch *et al*. note that ‘global health diplomacy seeks to facilitate global coordination and policy coherence for health’ (Kickbusch 2022). However, recognizing the ‘highly interconnected’ and ‘cascaded nature’ of a polycrisis (Kwamie et al. 2024), Hocking and colleagues advocate for ‘integrative diplomacy’, which emphasizes cross-sectoral collaboration, multi-level engagement, and whole-of-government approaches (Hocking et al. 2012). Applying this concept to global health, integrative health diplomacy may be better suited to coherently address overlapping public health threats by providing a framework to harmonize competing agendas and align fragmented governance mechanisms. In this context, strengthening coherence between GHS and UHC can be a foundational pillar for integrative health diplomacy, unifying diplomatic strategies across the spectrum from disease prevention to emergency response.

Informants highlighted that fragmentation between GHS and UHC has resulted in disjointed global health commitments and funding streams, leaving critical health initiatives vulnerable to backsliding and budget cuts. Increasing isolationism further threatens global health and development assistance, with hard power politics gaining traction in foreign policy circles (Hocking et al. 2012, Holmer 2024, Rasanathan 2024). GHS–UHC coherence offers a necessary counterweight to these trends, providing a strategic pathway to sustain progress amid geopolitical instability and the turbulence of a polycrisis. A hybrid norm integrating GHS’s security imperatives with UHC’s universal access principles ensures that global health agreements retain a balanced focus on resilience to emergencies without compromising equity in care delivery (Lal et al. 2022b). For instance, coherence between both norms may better enable emergency response strategies that invest in PHC infrastructure in climate-affected regions, aligning outbreak control with long-term service continuity and health system resilience. As global governance tilts toward defense-driven priorities, the emerging GHS–UHC hybrid norm can prevent a hypersecuritized global health agenda, ensuring equity remains central to health policy and planning efforts.

Recent negotiations have already reflected this normative shift, with UHC norms like accessible countermeasures embedded in the draft Pandemic Agreement and GHS priorities like outbreak surveillance reinforced in the 2023 UHC Political Declaration (Lal et al. 2024b). This form of integrative health diplomacy, building on GHS and UHC coherence, will be crucial to both agendas, ensuring that neither is sacrificed to austerity or geopolitical interests. This not only streamlines duplicative health negotiations related to national security and human rights but also creates a unified narrative for national and multilateral health obligations and improves the ability of governments to support pooled global health financing mechanisms.

The pursuit of a GHS–UHC hybrid norm strengthens global health diplomacy by creating a shared framework for negotiation across sectors, governance levels, and geopolitical divides. Integrative diplomacy, as Hocking *et al*. assert, ‘can strengthen a country’s role as a negotiating partner in the bilateral and global arena’ (Hocking et al. 2012). For example, by jointly embedding equity and resilience as core principles, GHS–UHC coherence enables countries to balance global commitments to pandemic preparedness with local commitments to reduce air pollution and improve water sanitation, crucial to addressing the climate–health interface (Quintana et al. 2024). This alignment enhances both chronic and acute public health responses (Hanefeld et al. 2018, Haldane and Morgan 2021, Mustafa et al. 2022), equipping states with improved capacity to engage in effective health diplomacy particularly where domestic health issues intersect with international obligations -- thus improving crisis coordination and establishing enduring frameworks for joint action on broader health and development challenges. In an era where multilateral solidarity is increasingly fragile, this coherence between GHS and UHC offers an alternative to the rise of transactional diplomacy, ensuring that global health remains a pillar of cooperative international engagement rather than a casualty of shifting political tides.

## Conclusion

This study addresses chronic fragmentation across the global health architecture by examining how greater normative coherence between GHS and UHC can strengthen global health diplomacy, governance, financing, and health systems. Positioned along a continuum from prevention to response, GHS and UHC encompass a wide spectrum of global health priorities, making their integration not just a theoretical exercise but a necessary evolution for health policy and planning. While previous research has analyzed the construction and convergence of GHS and UHC norms, this study is among the first to directly assess the perspectives of key stakeholders engaged in efforts to improve their coherence. Of note, this paper does not claim that a fully-formed hybrid norm linking GHS and UHC already exists. Rather, it explores whether and how such a norm may be in the making, and what its emerging contours might look like based on stakeholder perspectives. These revisions help distinguish between the study of norm interaction and the potential crystallization of a new, distinct ‘hybrid norm’ over time.

The interviews reveal that actors’ conceptualizations of GHS and UHC have evolved post-pandemic. Equity and community health services are increasingly emphasized within GHS norms, while UHC norms were framed as essential for resilience despite recent political deprioritization. Respondents generally recognize both frameworks as closely interlinked through health systems. Factors influencing coherence include strategic motivations such as improving health outcomes and maximizing efficiencies. However, concerns such as internal resistance and weakened messaging persist. Structural barriers like conceptual misalignment and power imbalances hinder progress, while enablers like external support and robust internal implementation facilitate better integration. Important variations in actor perspectives across institutional and geopolitical context highlight both tensions and opportunities for advancing GHS–UHC coherence.

By examining what different actor groups think about GHS–UHC coherence—what it means in practice, how it can be achieved, and which challenges remain—this study offers a crucial window into the politics shaping GHS–UHC integration, and provides actionable insights into how coherent norms can advance these synergies in a post-pandemic context. The findings demonstrate that promoting this emerging hybrid norm provides a concrete pathway to bridging divides between securitized, international cooperation-driven approaches and rights-based, community-centered approaches. This is achieved by leveraging the complementary strengths, underlying principles, and core capacities of both frameworks. Intentional operationalization of coherence is not just about aligning policies and reconciling divergent health strategies among key actors—it is about safeguarding hard-won gains against competing priorities, ensuring neither agenda is sacrificed amid shifting geopolitical dynamics. In an era of rising austerity, eroding multilateralism, and increasing contestation, mainstreaming GHS–UHC coherence counters the pattern of continued fragmented global health efforts by providing a more pragmatic, unified approach to strengthening health systems for future crises.

Having unpacked how different global health stakeholders conceptualize, negotiate, and implement GHS–UHC norms, this research sheds light on the political and institutional processes required to strengthen coherence across global health initiatives. However, future research is needed to better assess the effectiveness of GHS and UHC framings among different stakeholders (Akhavein et al. 2025), as well as how normative coherence is implemented in national and subnational contexts.

Beyond the immediate policy implications, this study contributes to broader global health scholarship by positioning normative coherence as a critical factor in diplomacy, governance, and institutional design. Previous efforts to improve integration have often failed due to an insufficient focus on the politics of global health norms. As this study illustrates, coherence is not merely a technical or administrative function—it is a strategic and political endeavor. As the global health landscape evolves in an era of polycrisis and significant aid cuts, recognizing the inevitability of overlapping health agendas is paramount. Addressing fragmentation requires solutions as interconnected as the challenges they seek to resolve. The hybrid norm of GHS–UHC coherence provides a compelling framework for integrating previously siloed principles and core capacities, ultimately helping to address a wide range of global health challenges. This approach is also crucial to better enabling cross-sectoral collaboration, particularly between policy agendas that have been challenging to integrate, such as One Health, planetary health, or the humanitarian–development–peace nexus (Talisuna et al. 2025). Normative integration between both GHS and UHC – grounded in shared principles of equity, resilience, and accountability – must be treated as a core objective of financing and governance reforms moving forward. Without it, the ambition to foster integration will remain aspirational, and fragmentation will persist despite earnest efforts. This is vital to improving coherence across the global health architecture.

While this study centres on the synergies between GHS and UHC, its broader objective is to foster a more equitable and resilient global health architecture by tackling interconnected causes of fragmentation through GHS–UHC coherence. By advancing mutually-reinforcing solutions across governance and diplomacy mechanisms through hybrid normative frameworks, this work reimagines how global health institutions collaborate, ultimately helping to develop a more adaptive, inclusive, and sustainable approach to global health—one that is better equipped to navigate future political, economic, and social challenges.

## Supplementary Material

czaf086_Supplementary_Data

## Data Availability

The data underlying this article cannot be shared publicly due to privacy of individuals that participated in the study. The data will be shared on reasonable request to the corresponding author.

## Reflexivity statement

As an early-career researcher based in a Global North academic institution, I recognize the importance of inclusivity in global health research. While I was the sole author of this study, as it represents the final installment of my doctoral research, it has been deeply influenced by collaboration with diverse scholars across disciplines and geographies. I drew from the collective wisdom of global health practitioners, policymakers, and academics, ensuring that perspectives from the Global South were central to the analysis. As a child of South Asian immigrants with an intersectional background, I am acutely aware of structural inequities in global health research and the need to elevate underrepresented voices. This study sought to do so by centring insights from a wide range of stakeholders, particularly those from low- and middle-income countries. Moving forward, I remain committed to fostering equitable collaborations and amplifying diversity and inclusion in global health policy and governance.
