# Supplementary material for: Towards a coherent global health architecture: perspectives on integrating global health security and universal health coverage through diplomacy and governance reforms
Source: Health Policy Plan. 2025 Oct 31;41(2):162–75. doi: 10.1093/heapol/czaf086 (PMC12906769; doi:10.1093/heapol/czaf086)
Supplement: czaf086_Supplementary_Data [file czaf086_supplementary_data.zip › Pathways to coherence - Table 1.docx]

**Table 1.** Number of key informants interviewed per actor grouping.

| **Actor Type** | **# of participants** |
| --- | --- |
| *Multilateral and global health organizations* | **9** |
| *Country governments* | **10** |
| *Donors, foundations, and international finance institutions* | **6** |
| *Civil society organizations* | **6** |
| ***Total*** | ***31*** |
