# Supplementary material for: Towards a coherent global health architecture: perspectives on integrating global health security and universal health coverage through diplomacy and governance reforms
Source: Health Policy Plan. 2025 Oct 31;41(2):162–75. doi: 10.1093/heapol/czaf086 (PMC12906769; doi:10.1093/heapol/czaf086)
Supplement: czaf086_Supplementary_Data [file czaf086_supplementary_data.zip › Pathways to coherence - Table 3.docx]

**Table 3.** Findings from thematic analysis of interview transcripts, organized by category, theme, and subtheme.

| **Categories** | **Themes** | **Subthemes** |
| --- | --- | --- |
| **Evolving perceptions of GHS, UHC, and their relationship** | *Definitional shifts* | - GHS emphasis on equity and community-level health services - UHC as key to resilience, despite recent deprioritization |
|  | *Conceptual relationships* | - UHC as foundation for GHS - Interlinked through health system |
| **Factors influencing coherence** | *Strategic considerations* | Motivations   - Improving health outcomes - Maximizing efficiencies - Sustaining progress for both   Concerns   - Harder to demonstrate progress - Internal resistance and competition - Weakened messaging |
|  | *Structural considerations* | Barriers   - Conceptual and structural misalignment - Power dynamics & external influences - Resource allocation & accountability   Enablers   - Clear messaging - External support - Internal implementation |
